# Influence of Genetic Polymorphisms on Cognitive Function According to Dietary Exposure to Bisphenols in a Sample of Spanish Schoolchildren

**DOI:** 10.3390/nu16162639

**Published:** 2024-08-10

**Authors:** Viviana Ramírez, Patricia González-Palacios, Pablo José González-Domenech, Sonia Jaimez-Pérez, Miguel A. Baca, Lourdes Rodrigo, María Jesús Álvarez-Cubero, Celia Monteagudo, Luis Javier Martínez-González, Ana Rivas

**Affiliations:** 1 Department of Nutrition and Food Science, Faculty of Pharmacy, University of Granada, 18071 Granada, Spain; vivianarl@ugr.es (V.R.); pgonzapa@ugr.es (P.G.-P.); amrivas@ugr.es (A.R.); 2 GENYO Centre for Genomics and Oncological Research, Pfizer/University of Granada/Andalusian Regional Government PTS Granada—Avenida de la Ilustración, 114, 18016 Granada, Spain; luisjavier.martinez@genyo.es; 3 Instituto de Investigación Biosanitaria ibs.GRANADA, 18012 Granada, Spain; 4 Institute of Nutrition and Food Technology “Jose Mataix Verdú”, Biomedical Research Center, Health Sciences Technological Park, University of Granada, 18016 Granada, Spain; 5 Department of Psychiatry, Faculty of Medicine, University of Granada, 18012 Granada, Spain; pgdomenech@ugr.es; 6 Virgen de las Nieves University Hospital, 18014 Granada, Spain; soniajaimezperez@gmail.com; 7 Clinica MenSana, 18009 Granada, Spain; mabaca@cop.es; 8 Department of Legal Medicine, Toxicology and Physical Anthropology, Faculty of Medicine, University of Granada, 18012 Granada, Spain; lourdesr@ugr.es; 9 Department of Biochemistry and Molecular Biology III, Faculty of Medicine, University of Granada, 18012 Granada, Spain

**Keywords:** cognitive function, neurodevelopmental disorders, genetic polymorphism, dietary exposure, bisphenols

## Abstract

Background: Neurodevelopmental disorders (NDDs) like intellectual disability (ID) are highly heritable, but the environment plays an important role. For example, endocrine disrupting chemicals (EDCs), including bisphenol A (BPA) and its analogues, have been termed neuroendocrine disruptors. This study aimed to evaluate the influence of different genetic polymorphisms (SNPs) on cognitive function in Spanish schoolchildren according to dietary bisphenol exposure. Methods: A total of 102 children aged 6–12 years old were included. Ten SNPs in genes involved in brain development, synaptic plasticity, and neurotransmission (*BDNF*, *NTRK2*, *HTR2A*, *MTHFR*, *OXTR*, *SLC6A2*, and *SNAP25*) were genotyped. Then, dietary exposure to bisphenols (BPA plus BPS) was estimated and cognitive functions were assessed using the WISC-V Spanish form. Results: *BDNF* rs11030101-T and *SNAP25* rs363039-A allele carriers scored better on the fluid reasoning domain, except for those inheriting the *BDNF* rs6265-A allele, who had lower scores. Secondly, relevant SNP–bisphenol interactions existed in verbal comprehension (*NTRK2* rs10868235 (*p*-int = 0.043)), working memory (*HTR2A* rs7997012 (*p*-int = 0.002), *MTHFR* rs1801133 (*p*-int = 0.026), and *OXTR* rs53576 (*p*-int = 0.030)) and fluid reasoning (*SLC6A2* rs998424 (*p*-int = 0.004)). Conclusions: Our findings provide the first proof that exploring the synergistic or additive effects between genetic variability and bisphenol exposure on cognitive function could lead to a better understanding of the multifactorial and polygenic aetiology of NDDs.

## 1. Introduction

DSM-V (Diagnostic and Statistical Manual of Mental Disorders, fifth edition) defines neurodevelopmental disorders (NDDs) as a heterogenous group of mental health conditions that occur during the developmental period and negatively affect brain functioning [1]. NDDs include attention-deficit hyperactivity disorder (ADHD), autism spectrum disorder (ASD), and intellectual disability (ID), which lead to behavioural problems, poorer learning, memory dysfunction, and delayed motor development [2,3]. Among these, cognitive impairments in general and ID in particular constitute major conditions of NDDs with diverse aetiologies, affecting about 1% of children in the world [4,5]. They are characterised by both impaired cognitive functioning (intellectual quotient (IQ) < 70) and adaptive behaviour [6].

Non-genetic causes such as infections, autoimmunity, and environmental factors are described in NDD pathogenesis, but advances in biomolecular knowledge (e.g., genotyping/sequencing approaches) have identified hundreds of candidate genes to be involved in neurodevelopment, revealing the importance of a genetic contribution [7,8]. In fact, ID has emerged as the most common manifestation under genetic abnormalities [5,9]. Structural variants such as copy number variations (CNVs) and point mutations like single nucleotide variants (SNVs) have been found in patients suffering from neurodevelopmental alterations [10]. In certain cases, one single de novo mutation could be the causative factor, while in other scenarios, the risk of developing NDDs could be influenced by a complex interplay between rare and common genetic variants [7]. Specifically, single nucleotide polymorphisms (SNPs), which are common SNVs occurring with a frequency of at least 1%, have shown to contribute to mild intellectual impairment [11]. Brain-derived neurotrophic factor (*BDNF*) rs6265 (Val66Met) is one of the most extensively studied missense variants within the prodomain region of BDNF, with functional consequences on memory, cognition, and behaviour [12].

Although the identification of NDD-causing genes is essential for understanding the underlying biological mechanisms responsible for the onset of these disorders, the molecular diagnosis is quite challenging and still unknown in many patients [2]. This highlights the complex and multifactorial nature of NDDs and the need to examine other risk factors at the same time. Endocrine disrupting chemicals (EDCs) such as bisphenol A (BPA) and its analogues are able to cross the blood–brain barrier and, as the developing brain is particularly sensitive to these compounds, EDCs have been termed neuroendocrine disruptors [13]. BPA migration from food packaging into foodstuffs is a significant contamination source by which BPA enters the food chain, and for this reason, dietary consumption has been considered the primary contributor to BPA exposure, followed by contaminated air and dermal absorption [14]. To date, BPA exposure during childhood has been more frequently related to adverse behavioural outcomes, whereas evidence for effects on cognitive functioning is still weak [15,16]. For this reason, the exploration of the synergistic or additive effect between the environmental factor and genetic vulnerability could lead to a better understanding of the multifactorial and polygenic aetiology of NDDs [17,18]. To the best of our knowledge, there is growing evidence of interactions between gene polymorphisms and pesticides/heavy metals in cognitive development and the etiopathogenesis of disorders such as ASD and ADHD [10]; nonetheless, no human studies examining NDD-associated genetic variants in the presence of bisphenol exposure are available.

Therefore, the purpose of the current study was to evaluate the influence of different genetic polymorphisms on cognitive function in Spanish schoolchildren aged between 6 and 12 years according to dietary exposure to bisphenols.

## 2. Materials and Methods

### 2.1. Study Subjects and Data Collection

Participants enrolled in this study were recruited from different elementary schools and health centres in Granada, Spain, between 2020 and 2023 as part of a larger research project. Inclusion criteria for the selection of the study population were (1) schoolchildren aged between 6 and 12 years, and (2) having lived in the study area for at least 6 months continuously. Children whose parents or legal tutors agreed to participate and signed the written informed consent form were contacted by the paediatric clinical centre specialised in neurodevelopmental disorders. The study protocol was approved by the Ethics Committee of Provincial Biomedical Research of Granada (1742-N-23).

A total of 102 children with available estimates of dietary exposure to bisphenols, good quality DNA samples, and neurodevelopmental tests assessing cognitive function were finally selected for the current study.

Face-to-face interviews were conducted with all participants’ parents or guardians by trained interviewers. The structured questionnaire was based on a sociodemographic section (gender and age of children and educational level, occupational rank, and marital status of parents or legal guardians), lifestyles (physical and dietary patterns) and anthropometric data collected by qualified personnel (weight and height).

### 2.2. DNA Isolation and Genotyping Assays

For genotyping, DNA was extracted from buccal swabs using a procedure based on proteinase K digestion and saline purification. DNA quantification was performed using the Qubit^TM^ 4.0 fluorometer (Invitrogen^TM^ by ThermoFisher Scientific, Waltham, MA, USA) with the Qubit dsDNA BR Assay Kit (Invitrogen^TM^ by ThermoFisher Scientific, Hillsboro, OR, USA). DNA samples were frozen at −20 °C until the genotyping step.

Ten SNPs were selected based on two selection criteria: (1) a minor allele frequency (MAF) higher than 10% within the Iberian population and (2) a greater number of studies on the association with neurodevelopmental functions in healthy and clinical populations. These SNPs are in genes involved in brain development and synaptic plasticity (*BDNF* rs6265 and rs11030101; neurotrophic receptor tyrosine kinase 2 (*NTRK2*) rs2289656 and rs10868235; methylenetetrahydrofolate reductase (*MTHFR*) rs1801133; and synaptosome associated protein 25 (*SNAP25*) rs363039) and neurotransmitter systems (5-hydroxytryptamine receptor 2A (*HTR2A*) rs6314 and rs7997012; oxytocin receptor (*OXTR*) rs53576; and solute carrier family 6 member 2 (*SLC6A2*) rs998424).

Information on the gene, chromosomal location, variant effect, genotype, and allele frequencies were obtained from Ensembl “https://www.ensembl.org/index.html (accessed on 22 January 2024)”and The National Centre for Biotechnology Information SNP website “https://www.ncbi.nlm.nih.gov/ (accessed on 22 January 2024)”, and are listed in Table 1.

Two types of genotyping technologies were performed: (1) Infinium Global Screening Array (GSA)-24 BeadChip and (2) Taqman SNP Genotyping Assays. In the first place, 7 SNPs were genotyped using the microarray technology on the iScan system by Ilumina^®^ Infinium^®^ HTS Assay (Illumina, Inc., San Diego, CA, USA) according to the method previously described by Ramírez et al. (2023) [19]. GSA data were read and analysed with the software llumina^®^ GenomeStudio v2010.3.

In Taqman assays, 3 SNPs were genotyped by the following commercially available Taqman^®^ probes (Applied Biosystems™ Taqman SNP Genotyping Assays): C___1751785_10 for *BDNF* rs11030101, C___3020067_10 for *SLC6A2* rs998424, and C____327976_10 for *SNAP25* rs363039. Quantitative PCRs (qPCRs) were performed on the QuantStudio™ 6 Flex Real-Time PCR System (Applied Biosystems™, Waltham, MA, USA) and data outputs were read and processed with the software QuantStudio™ Real-Time PCR v1.3.1 [20]. 

Those SNPs presenting a call rate of less than 95% that deviated from Hardy–Weinberg equilibrium (HWE, *p* < 0.05) and samples with an overall call rate of less than 95% were excluded from the final statistical analysis.

### 2.3. Bisphenol Exposure Assessment

Daily dietary exposure to total bisphenols (BPA plus BPS) was estimated on an individual basis by multiplying the daily intake of different foods (g/day) by the corresponding bisphenol content in each food item (ng/g of food). The dietary information was recorded for the last 12 months through a semi-quantitative food frequency questionnaire (FFQ). This food survey was designed to ask about the food frequency (g of food per day) of 112 food items categorised into 13 groups, e.g., dairy products, meat and meat products, vegetables, legumes, and cereals, among others [21]. After that, the bisphenol content was chemically determined via an ultra-high performance liquid chromatography–tandem mass spectrometry (UHPLC-MS/MS) system following the methodology described by Galvez-Ontiveros et al. (2021) [22]. Finally, BPA intake from all food sources analysed was summed for all individuals to estimate the total exposure dose (ng/day).

### 2.4. Neurodevelopmental Assessment

Cognitive functions in children aged 6–12 years were assessed using the Spanish form of the Weschler Intelligence Scale for Children—Fifth Edition (WISC-V), administrated by licensed and trained psychologists in childhood neurodevelopment. The WISC-V assesses various cognitive domains, providing a comprehensive profile of a child’s cognitive abilities. The test is composed of 10 primary subtests, which can be combined into composite quotients, yielding five age-standardised primary indices: Verbal Comprehension Index (VCI), Visual Spatial Index (VSI), Fluid Reasoning Index (FRI), Working Memory Index (WMI), and Processing Spead Index (PSI). The Full-Scale Intelligence Quotient (FSIQ) is derived from seven primary subtests, typically Similarities, Vocabulary, Block Design, Matrix Reasoning, Figure Weights, Digit Span, and Coding.

For this study, the five primary indices and FSIQ scores (mean = 100, standard deviation (SD) = 15) were selected to address the cognitive profiles and IQ.

### 2.5. Data Analysis

Descriptive analyses of quantitative variables were carried out using the means and SDs for parametric variables, and medians and interquartile ranges (IQRs) in the case of non-parametric variables. The qualitative variables are presented in terms of frequencies and percentages. The Kolmogorov–Smirnov test with Lilliefors correction was performed to check the normality of continuous data.

To assess Hardy–Weinberg equilibrium (HWE), chi-square tests were applied (*p* > 0.05) in the codominant model. Linkage disequilibrium (LD) analyses were performed using SNPStats software “https://snpstats.net/start.htm (accessed on 10 February 2024)”. SNPs were in LD when they had an r^2^ value higher than 0.5. After verifying HWE and LD, the analyses were undertaken within the dominant or recessive model, and the contribution per allele was tested.

Student’s *t*-test and the Mann–Whitney test were conducted for parametric and non-parametric variables, respectively. They were used to compare WISC-V index scores for each different genetic variant.

Crude odds ratios (ORs) and 95% confidence intervals (95% CIs) were calculated using binary logistic regression models to evaluate the influence of the genetic variants on WISC-V index scores. The WISC-V index was entered as the dependent variable, each genetic polymorphism as the independent variable, and dietary exposure to bisphenols (stratified by low and high exposure according to median values expressed as ng/day) was input as the selecting variable. Multivariable logistic regression models were then fitted that included sex, age, body mass index (BMI), and/or parental education level as potential confounders of neurological testing [23,24]. Sex and age were used as confounding factors in all analyses, and BMI and parental education level were included in the model if they produced changes in the OR of more than 10%. To explore gene–environment interactions in cognitive functions, the interaction term “polymorphism x exposure level” was added to the logistic regressions. Statistical significance was based on a *p* value ≤ 0.05. In addition, Bonferroni’s correction was applied to the multifactorial logistic regression *p* values to account for the multiple testing of 10 different SNPs (*p* ≤ 0.005). All statistical analyses were performed with IBM SPSS Statistics 25 (Armonk, NY, USA) and RStudio 2023.12.0.

## 3. Results

### 3.1. Characteristics of Participants

Baseline characteristics of the study population are shown in Table 2. Of the 102 children included, 53 (52%) were boys and the mean age was 8.7 ± 2.1 years. The estimated daily dietary exposure dose for total bisphenols was 17306.3 ng/day. The educational level of the parents was classified into primary, secondary, and university education, with most of parents belonging to university category (50%). Regarding overall cognitive performance, the mean of the WISC-V FSIQ was 101.1 (12.7).

### 3.2. Genetic Variants and WISC-V Scores

All SNPs achieved HWE (*p* > 0.05, Table 1). The MAFs of each locus were in agreement with those established for the Iberian population; only for NTRK2 rs10868235 G/A was the variant A allele the minor allele in our cohort instead of the previously reported reference G allele. Those SNPs within the same gene were not in LD (BDNF rs6265/rs11030101 r^2^ = 0.17; HTR2A rs6314/rs7997012 r^2^ = 0.06; and NTRK2 rs2289656/rs10868235 r^2^ = 0.04).

Table 3 shows in detail the mean and median values of the WISC-V index scores obtained for each genetic variant. For the first BDNF rs6265/rs11030101 variant pair, opposite effects were found. Children with BDNF rs6265 AG/AA genotypes had significantly lower FRI scores than those homozygous for the reference G allele (*p* = 0.030). On the contrary, children who carried one or two copies of the rs11030101 minor T allele displayed significantly higher FRI (*p* = 0.009) scores than children who showed the wild AA genotype, suggesting a protective effect.

This protective trend was maintained for other genetic variants. For example, children inheriting at least one copy of the variant allele of *MTHFR* rs1801133 G/A and *SNAP25* rs363039 G/A obtained better scores on the visual spatial (*p* = 0.038 for rs1801133), verbal comprehension, and fluid reasoning domains (*p* = 0.026 and *p* = 0.004 for rs363039, respectively).

**Figure 1:**
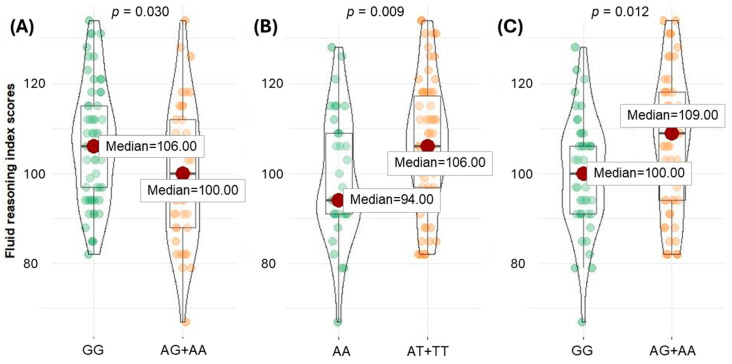
Fluid reasoning index scores obtained for (**A**) *BDNF* rs6265, (**B**) *BDNF* rs11030101, and (**C**) *SNAP25* rs363039.

Looking at these results, more significant differences in fluid reasoning scores were observed under the dominant model of *BDNF* rs6265/rs11030101 and *SNAP25* rs363039 variants (Figure 1).

### 3.3. Influence of Genetic Variants on the Cognitive Profile Assessed by WISC-V According to Dietary Exposure to Bisphenols

Here, the contribution of each genetic variant to possible changes in cognitive function was addressed by dividing the population into groups with low and high exposure to bisphenols. When the dietary exposure factor was entered, highly significant associations between genetic polymorphisms and WISC-V indices were obtained, which were even stronger after adjustment for sex, age, BMI, and/or parental education levels as covariates. The SNP-by-bisphenol exposure interaction was also explored to verify if the effect of the variant depended on the magnitude of exposure. Table 4 shows only the significant outcomes; the rest of the results are fully described in the Supplementary Material (Table S1).

Focusing on SNP pairs for *BDNF* and its receptor *NTRK2*, the *BDNF* rs11030101 variant T allele conferred protection against verbal comprehension dysfunction (adjusted OR = 0.26, *p* = 0.011, *p* interaction = 0.067).

*NTRK2* SNPs showed a dual effect, where the rs2289656 G/A polymorphism proved to be a risk variant (adjusted OR = 6.72, *p* = 0.004, *p* interaction = 0.062 for VCI), whereas rs10868235 developed a protective function in two cognitive aspects (adjusted OR = 0.22, *p* = 0.062, *p* interaction = 0.043 for VCI; and adjusted OR = 0.18, *p* = 0.034, *p* interaction = 0.020 for VSI), and the interaction was significant.

With regards to the serotonin signalling pathway, two variants within the *HTR2A* gene were explored and, once again, opposite associations were observed. The rs6314 G/A polymorphism seemed to confer a protective effect on poorer verbal comprehension at low exposure (adjusted OR = 0.15, *p* = 0.042, *p* interaction = 0.820) In contrast, the rs7997012 A/G effect differed based on the exposure degree: a significant decline in working memory was appreciated at low exposure levels (adjusted OR = 6.30, *p* = 0.017), whereas a modest improvement was observed at high levels (adjusted OR = 0.27, *p* = 0.096). After Bonferroni’s correction, strong interaction evidence (*p* interaction = 0.002) resulted from this differential effect.

For the *MTHFR* rs1801133 G/A polymorphism in the per-allele contribution model, the presence of the variant A allele was associated with a reduced likelihood of cognitive dysfunctions than the presence of the reference G allele at a low exposure dose (adjusted OR = 0.28, *p* = 0.010, *p* interaction = 0.026 for WMI; and adjusted OR = 0.36, *p* = 0.030, *p* interaction = 0.025 for FSIQ).

Lastly, a protective role was observed for the genetic variants *OXTR* rs53576 A/G (adjusted OR = 0.08, *p* = 0.007, *p* interaction = 0.030 for WMI) and *SLC6A2* rs998424 G/A (adjusted OR = 0.16, *p* = 0.005, *p* interaction = 0.004 for FRI) in terms of high exposure. After Bonferroni’s correction, the association and interaction persisted for *SLC6A2* rs998424. Other genetic variants, such as *SNAP25* rs363039 G/A, maintained their remarkable protective function independently of the exposure, resulting in a non-significant interaction (*p* interaction of 0.258, 0.775, and 0.378 for FRI, WMI and FSIQ, respectively).

**Figure 2:**
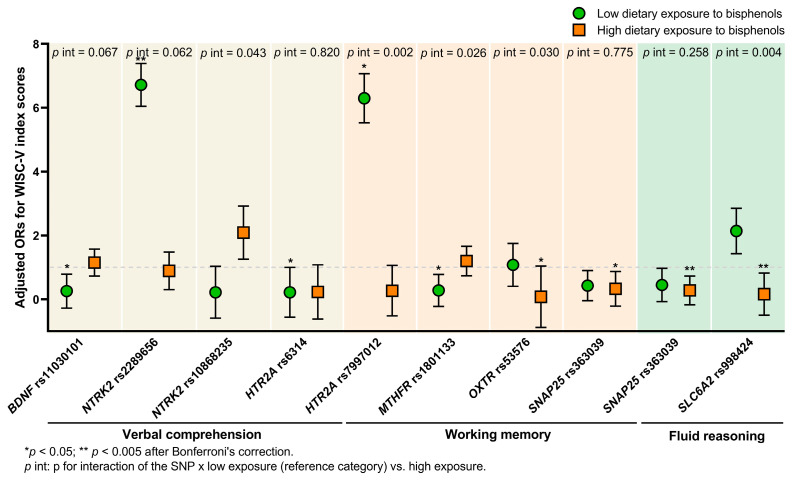
Influence of genetic polymorphisms on specific cognitive domains based on the level of bisphenol exposure.

Figure 2 highlights the associations and interactions obtained mainly for the verbal comprehension, working memory, and fluid reasoning domains.

## 4. Discussion

As far we know, our findings suggest for the first time that neurodevelopment-related gene polymorphisms play an important role in cognition measured through WISC-V in Spanish children exposed to dietary bisphenols. The main outcomes of the current research included the following aspects: (1) significant differences in fluid reasoning scores were observed mainly for *BDNF* rs6265/rs11030101 and *SNAP25* rs363039 variants, and (2) consistent associations of *BDNF* rs11030101, *NTRK2* rs2289656/rs10868235, *MTHFR* rs1801133, *HTR2A* rs7997012, *OXTR* rs53576, and *SLC6A2* rs998424 with certain cognitive domains and global intelligence index were obtained in the presence of bisphenol exposure, resulting in relevant SNP–bisphenol interactions.

Gene polymorphisms selected for this study are located in genes responsible for key neurodevelopmental processes, and it is well known that NDDs such as ADHD, ASD, and ID are genetically linked through common genetic alterations [6].

*BDNF* and its receptor tropomyosin receptor kinase B (TrkB), encoded by the *NTRK2* gene, are an essential regulatory system for neuronal development, synaptogenesis, and plasticity [25]. The possible involvement of *BDNF* in cognitive dysfunction was observed in children with ID showing reduced BDNF protein levels [26]. Furthermore, it has been evidenced that *BDNF* and *NTRK2* variants are associated with changes in hippocampal volume and altered performance on learning and memory tasks [25,27]. *BDNF* rs6265 (Val66Met) is one of the most extensively studied missense variants within the prodomain region of *BDNF*, with functional consequences for memory, cognition, and behaviour [12].

In our study, rs6265 variant A allele carriers had lower scores on the fluid reasoning domain, whereas children with the rs11030101 T allele experienced a better scenario for this cognitive component. These polymorphisms have been reported to be associated with other psychiatric and neurological disorders like major depressive disorder (MDD) [28,29], schizophrenia, or epilepsy [30]. However, no associations were found with cognitive outcomes [31,32].

Our gene–environment association analysis revealed interactions between variants in the *BDNF-NTRK2* system, such as rs10868235, and exposure to bisphenols in the context of verbal comprehension and visual spatial skills. Although there are no studies assessing interactions between these SNPs and dietary contaminants in neurodevelopment, some evidence suggests that BPA may interfere with the *BDNF* signalling pathway, leading to behavioural and cognitive impairments [33,34].

Like the *BDNF-NTRK2* system, *MTHFR* and *SNAP25* are involved in brain development and synaptic plasticity, respectively [35,36]. Firstly, proper folate metabolism is required for normal brain development, and so disruptions in this process may contribute to neurological disorders [35]. MTHFR is a key folate metabolism enzyme, whose deficiency has been correlated with common variants like rs1801133 (C677T) and rs1801131 (A1298C) [37]. We found that the presence of the variant A allele of the rs1801133 G/A polymorphism was linked to higher scores for working memory and FSIQ at a low bisphenol exposure dose (Table 4). This finding makes sense given the peculiar U-shaped dose–response curve followed by bisphenols, indicating the importance of investigating effects at both low and high exposure levels. In line with our result, the rs1801133 A allele was also found to attenuate the negative effect of *COMT* Val homozygosity on IQ in patients with schizophrenia [38]. A meta-analysis by Sun et al. (2021) did not find associations between this *MTHFR* SNP and mild cognitive impairment [39]. At the level of gene–environment interactions, possible connections of bisphenols with disrupted *MTHFR* metabolic functions have not yet been established.

For its part, the *SNAP25* gene is involved in synaptic plasticity, neuronal maturation, and neurotransmission [36]. In children with borderline intellectual functioning, *SNAP25* polymorphisms were associated with lower scores for the perceptual reasoning index and FSIQ [36]. In the present child population, the *SNAP25* rs363039 G/A variant maintained its protective function in fluid reasoning, working memory, and overall IQ, independent of the exposure. In agreement with this finding, the A allele of rs363039 was reported to be beneficial for working memory in individuals with ADHD [40].

On the other hand, we have also focused on genetic changes at the level of neurotransmitter systems (*HTR2A, OXTR,* and *SLC6A2*). The serotonin 2A receptor, encoded by the *HTR2A* gene, is located in brain regions essential for learning and cognition. In fact, polymorphisms within this gene, such the rs6314 (His452Tyr), have been associated with altered memory processes [41]. Consistent with this, we found that the *HTR2A* rs7997012 A/G variant was related to altered working memory at low bisphenol exposure, whereas the opposite effect was modestly observed at high levels, resulting in a strong interaction. This finding shed light that genetics interact with a dynamic environment, leading to differential effects depending on the exposure level. Conversely, the other variant, *HTR2A* rs6314, maintained its protective role against poorer verbal comprehension independent of the exposure level. Until now, evidence from animal studies has demonstrated that mixtures of EDCs, including BPA, could impair mouse behaviour by modifying the brain expression of *Htr1a* and *Htr2a* [42].

Another variant that showed a protective effect on working memory was the *OXTR* rs53576 A/G polymorphism in children with high bisphenol levels. This polymorphism is located in the gene encoding the receptor for oxytocin, a neuromodulator involved in forming social, working, spatial, and episodic memory [43]. *OXTR* rs53576 has been proven to be associated with poorer social cognition in children but also with protective social traits, such as prosocial and empathic behaviour [44,45,46]. Meanwhile, the *OXTR* rs53576–bisphenol interaction found in our study could make sense from in vivo studies. Here, perinatal exposure to BPA, alone or in a mixture, alters oxytocin and *OXTR* expression in a sex- and region-specific manner [42,47].

Finally, *OXTR* rs53576 also showed protection for fluid reasoning, but the interaction was not significant. However, a strong interaction was obtained for the *SLC6A2* rs998424 G/A variant. Polymorphic variants in this gene coding for the norepinephrine transporter have been implicated in ADHD-related impairments, such as altered intrinsic brain activity, visual memory, and attention in children [48,49,50]. As aforementioned, BPA exposure may affect the serotonergic and oxytocin systems in the brain, but the effects on the norepinephrine system remain unclear.

One limitation of our study was the sample size. Although this is a limitation of several genetic association studies [36,45,51], the insightful findings of our small-scale study highlight the value of further larger studies to replicate and validate the results. It is well established that adverse neurodevelopmental effects of bisphenols are more pronounced in early age [13]. To date, evidence of the effects of childhood BPA exposure on cognitive function remain inconclusive [15,16]. One study addressed associations of urinary BPA concentrations with WISC-IV scores at different ages [15], while another study used age as an adjusting variable [16]. Given the limited sample size, it was not possible to perform an age-stratified analysis, but the regression models were adjusted for age to minimise potential confounding effects.

An additional limitation is that the particular effect of each SNP varies depending on which allele is designated as the “risk” allele. This is the reason why contradictory results can be obtained between different studies for the same genetic variant. Furthermore, the study design (neurodevelopment assessment tool, ethnic heterogeneity, and selected study population) could explain the inconsistencies between studies. There are several non-dietary sources of human exposure to bisphenols, which were not considered for the purpose of this study; however, the largest contribution to total exposure comes from food intake, accounting for more than 90%, confirming that a dietary exposure assessment is the first step in addressing the bisphenol-associated health problems [52].

The main strength of the current study lies in providing insightful evidence on the influence of genetic polymorphisms on childhood cognitive function in the presence of exposure to bisphenols. Firstly, carriers of the *BDNF* rs11030101 T and *SNAP25* rs363039 A alleles obtained better scores on the fluid reasoning domain, except for those inheriting the *BDNF* rs6265 A allele, who had lower scores. In comparison with previous WISC versions, in WISC-V, the perceptual reasoning domain is divided into FRI and VSI, and the fluid reasoning domain could be a good indicator of intellectual functioning, as we have shown [53].

Secondly, we reported relevant SNP–bisphenol interactions in certain cognitive domains. Genetic variants in genes responsible for vital neurodevelopmental processes, such as brain development and synaptic plasticity (*BDNF* rs11030101, *NTRK2* rs2289656 and rs10868235, and *MTHFR* rs1801133) and neurotransmission (*HTR2A* rs7997012, *OXTR* rs53576, and *SLC6A2* rs998424) presented consistent associations with verbal comprehension, working memory, and fluid reasoning. The effects on these cognitive abilities depended on the level of exposure to bisphenol. Two aspects need to be highlighted here. (1) Genetics interact with an environment that is constantly changing, and for this reason the study of gene–environment interaction gives us a more complete answer to disease aetiology [54]; (2) EDCs, including bisphenols, follow a particular dose–response curve, with optimal effects at low doses, and so it is important to assess effects at low concentrations [55]. Additionally, (3) working memory is a cognitive domain involved in many aspects of neurodevelopment, and given the significance found in this area, we support considering the selected SNPs as genetic markers of cognitive alterations in individuals with NDDs. Similarly, the Weschler Intelligence Scales are the most widely used instruments for measuring cognitive function, and the latest version, the WISC-V, has undergone changes that may make it more reliable for assessing cognitive dysfunction in the etiopathogenesis of NDDs [53,56].

## 5. Conclusions

In conclusion, our findings demonstrate that SNPs related to brain development, synaptic plasticity, and neurotransmission are associated with differences in cognitive domains assessed by WISC-V, specifically fluid reasoning, verbal comprehension and working memory, in children exposed to bisphenols, revealing important SNP–bisphenol interactions. The exploration of gene–environment interactions could lead to a better understanding of the multifactorial and polygenetic aetiology of NDDs. For this reason, and in view of the lack of studies assessing the combined effects of genetic variability and exposure to bisphenols on cognitive function, we support considering them as interactive factors rather than individual contributors to NDDs.

## Figures and Tables

**Table 1:** Information on the selected SNPs in the Spanish reference population (N = 107) and in our cohort (N = 102).

Gene Name	Gene Function	rs ID	Chr Position (GRCh38/hg38)	Reference/Variant Allele	Variant Effect	MAF (N)		
						IBS ^a^	Our Cohort	HWE *p* Value ^b^
*BDNF*	Neuronal development, synaptogenesis, and plasticity	rs6265 (Val66Met)	chr11: 27658369	C/T or G/A	Missense variant	T: 0.210 (45)	A: 0.211 (43)	0.132
*BDNF*		rs11030101	chr11: 27659197	A/T	5 prime UTR variant	T: 0.435 (93)	T: 0.392 (80)	0.264
*HTR2A*	Learning and cognitive abilities	rs6314 (His452Tyr)	chr13: 46834899	G/A	Missense variant	A: 0.107 (23)	A: 0.103 (21)	0.324
*HTR2A*		rs7997012	chr13: 46837850	A/G	Intron variant	A: 0.388 (83)	A: 0.333 (68)	0.766
*MTHFR*	Brain development and synaptic plasticity	rs1801133 (C677T)	chr1: 11796321	G/A	Missense variant	A: 0.444 (95)	A: 0.377 (77)	0.823
*OXTR*	Social, working, spatial, and episodic memory formation	rs53576	chr3: 8762685	A/G	Intron variant	A: 0.308 (66)	A: 0.294 (60)	0.384
*SLC6A2*	Mood, attention, and stress response regulation	rs998424	chr16: 55698034	G/A	Intron variant	A: 0.308 (66)	A: 0.377 (77)	0.536
*SNAP25*	Brain development and synaptic plasticity	rs363039	chr20: 10239848	G/A	Intron variant	A: 0.383 (82)	A: 0.328 (67)	0.653
*NTRK2*	Neuronal development, synaptogenesis, and plasticity	rs2289656	chr9: 84948647	G/A	Intron variant	A: 0.206 (44)	A: 0.181 (37)	0.273
*NTRK2*		rs10868235	chr9: 84878840	C/T or G/A	Intron variant	C: 0.486 (104)	A: 0.480 (98)	0.831

MAF: minor allele frequency. ^a^ IBS: Iberian population MAF values from the Ensembl database “https://www.ensembl.org/index.html (accessed on 22 January 2024)”. ^b^ HWE: Hardy–Weinberg equilibrium by the chi-square test.

**Table 2:** General characteristics of the study population (N = 102).

Age in years, mean (SD)	8.7 (2.1)
Gender, n (%)	
Boys	53 (52.0)
Girls	49 (48.0)
Weight in kg, mean (SD)	36.9 (15.0)
Height in cm, mean (SD)	134.8 (18.8)
BMI in kg/m^2^, mean (SD)	19.3 (4.9)
Bisphenols in ng/day, median (IQR)	17306.3 (9674.2–27067.7)
Bisphenol A	6823.7 (3575.9–12305.9)
Bisphenol S	6976.4 (3459.9–17472.7)
Parental education level, n (%)	
Up to primary	12 (11.8)
Secondary	38 (37.3)
University	51 (50.0)
Missing data	1 (0.9)
WISC-V indices	
Verbal Comprehension Index (VCI), median (IQR)	106.0 (95.0–113.0)
Visual Spatial Index (VSI), mean (SD)	102.5 (15.3)
Fluid Reasoning Index (FRI), median (IQR)	106.0 (94.0–115.0)
Working Memory Index (WMI), mean (SD)	101.9 (14.2)
Processing Spead Index (PSI), median (IQR)	86.0 (77.0–92.0)
Full-Scale Intelligence Quotient (FSIQ), mean (SD)	101.1 (12.7)

SD: standard deviation; BMI: body mass index; IQR: interquartile range; bw: body weight.

**Table 3:** Scoring of each WISC-V index by genetic variant.

	VCI ^a^			VSI ^b^		FRI ^a^		WMI ^b^		PSI ^a^		FSIQ ^b^	
	N	Median (IQR)	*p* Value	Mean (SD)	*p* Value	Median (IQR)	*p* Value	Mean (SD)	*p* Value	Median (IQR)	*p* Value	Mean (SD)	*p* Value
*BDNF* rs6265 (Dom)													
GG	61	108.0 (95.0–116.0)	0.444	102.6 (12.9)	0.920	106.0 (95.5–115.0)	**0.030**	101.3 (14.7)	0.605	83.0 (76.0–92.0)	0.251	102.1 (12.0)	0.338
AG + AA	41	106.0 (95.0–111.0)		102.3 (18.5)		100.0 (88.0–112.0)		102.8 (13.6)		89.0 (80.0–95.0)		99.6 (13.6)	
G	161	106.0 (95.0–114.5)	0.520	102.5 (14.5)	0.967	106.0 (94.0–115.0)	0.069	101.6 (14.4)	0.572	86.0 (77.0–92.0)	0.278	101.4 (12.4)	0.476
A	43	106.0 (95.0–111.0)		102.4 (18.3)		100.0 (88.0–112.0)		103.0 (13.5)		89.0 (80.0–95.0)		99.9 (13.6)	
*BDNF* rs11030101 (Dom)													
AA	35	103.0 (92.0–113.0)	0.119	100.5 (13.5)	0.355	94.0 (91.0–109.0)	**0.009**	101.4 (13.1)	0.805	86.0 (77.0–95.0)	0.753	97.8 (11.5)	0.056
AT + TT	67	108.0 (98.0–118.0)		103.5 (16.2)		106.0 (97.0–118.0)		102.2 (14.8)		83.0 (77.0–92.0)		102.8 (13.0)	
A	124	106.0 (95.0–113.0)	0.261	101.5 (14.8)	0.277	103.0 (91.0–112.0)	**0.014**	101.7 (13.7)	0.808	86.0 (77.8–95.0)	0.404	99.9 (12.5)	0.101
T	80	108.0 (95.8–118.0)		103.9 (16.1)		106.0 (97.0–117.3)		102.2 (15.0)		83.0 (77.0–92.0)		102.9 (12.6)	
*HTR2A* rs6314 (Dom)													
GG	83	103.0 (95.0–113.0)	0.117	102.4 (15.4)	0.960	106.0 (91.0–115.0)	0.433	102.4 (14.4)	0.507	86.0 (77.0–92.0)	0.812	100.6 (13.0)	0.425
AG + AA	19	111.0 (100.0–118.0)		102.6 (15.4)		106.0 (97.0–115.0)		99.9 (13.6)		83.0 (77.0–95.0)		103.2 (11.1)	
G	183	106.0 (95.0–113.0)	0.109	102.5 (15.4)	0.942	106.0 (94.0–115.0)	0.443	102.1 (14.3)	0.536	86.0 (77.0–92.0)	0.799	100.8 (12.8)	0.385
A	21	111.0 (103.0–115.5)		102.2 (15.2)		106.0 (98.5–113.5)		100.1 (13.3)		89.0 (77.0–95.0)		103.4 (10.7)	
*HTR2A* rs7997012 (Rec)													
AA + AG	56	108.0 (95.0–116.0)	0.718	103.9 (16.0)	0.310	106.0 (94.0–115.0)	0.167	102.3 (14.4)	0.739	83.0 (77.8–92.0)	0.741	102.0 (13.0)	0.426
GG	46	104.5 (98.0–113.0)		100.8 (14.5)		106.0 (91.0–112.0)		101.4 (14.1)		86.0 (77.0–95.0)		100.0 (12.3)	
A	68	108.0 (95.0–115.3)	0.734	104.6 (16.2)	0.160	106.0 (94.0–115.0)	0.202	101.9 (14.1)	0.992	83.0 (77.0–92.0)	0.858	102.7 (12.8)	0.215
G	136	106.0 (95.0–113.0)		101.4 (14.8)		106.0 (91.0–112.0)		101.9 (14.3)		86.0 (77.0–92.0)		100.3 (12.5)	
*MTHFR* rs1801133 (Dom)													
GG	39	106.0 (95.0–111.0)	0.218	98.5 (14.5)	**0.038**	103.0 (91.0–115.0)	0.177	100.4 (14.5)	0.388	86.0 (80.0–92.0)	0.354	98.4 (12.5)	0.087
AG + AA	63	108.0 (95.0–116.0)		104.9 (15.4)		106.0 (97.0–115.0)		102.9 (14.0)		83.0 (77.0–92.0)		102.8 (12.6)	
G	127	106.0 (95.0–113.0)	0.462	100.9 (15.5)	0.061	103.0 (91.0–115.0)	0.214	101.0 (14.4)	0.243	86.0 (77.0–92.0)	0.634	100.0 (12.8)	0.060
A	77	108.0 (95.0–116.0)		105.1 (14.7)		106.0 (97.0–113.5)		103.4 (13.7)		83.0 (77.0–93.5)		102.9 (12.3)	
*OXTR* rs53576 (Rec)													
AA + AG	53	103.0 (95.0–113.0)	0.283	103.0 (15.0)	0.709	103.0 (91.0–110.5)	0.078	100.8 (13.8)	0.435	83.0 (77.0–92.0)	0.941	99.6 (13.4)	0.202
GG	49	106.0 (98.0–118.0)		101.9 (15.8)		109.0 (94.0–115.0)		103.1 (14.7)		86.0 (77.0–92.0)		102.8 (11.7)	
A	60	106.0 (95.0–113.0)	0.806	104.5 (16.6)	0.215	103.0 (91.0–114.3)	0.318	101.1 (13.4)	0.606	86.0 (77.8–92.0)	0.863	100.6 (13.8)	0.694
G	144	106.0 (95.0–113.0)		101.6 (14.7)		106.0 (94.0–115.0)		102.2 (14.5)		86.0 (77.0–92.0)		101.3 (12.1)	
*SLC6A2* rs998424 (Dom)													
GG	41	100.0 (95.0–112.0)	0.211	102.1 (14.5)	0.862	103.0 (91.0–113.5)	0.363	100.7 (15.3)	0.494	83.0 (77.0–92.0)	0.368	99.7 (12.8)	0.362
AG + AA	61	108.0 (95.0–116.0)		102.7 (16.0)		106.0 (94.0–115.0)		102.7 (13.5)		86.0 (80.0–93.5)		102.0 (12.5)	
G	127	103.0 (95.0–113.0)	0.111	102.8 (15.2)	0.733	106.0 (91.0–112.0)	0.155	101.9 (14.7)	0.969	86.0 (77.0–92.0)	0.687	100.5 (12.8)	0.396
A	77	106.0 (96.5–116.0)		102.0 (15.6)		106.0 (94.0–116.5)		102.0 (13.4)		86.0 (77.0–95.0)		102.1 (12.4)	
*SNAP25* rs363039 (Dom)													
GG	45	103.0 (92.0–111.0)	**0.026**	102.1 (14.1)	0.825	100.0 (91.0–107.5)	**0.012**	99.7 (12.8)	0.166	86.0 (80.0–93.5)	0.512	98.8 (11.8)	0.096
AG + AA	57	108.0 (98.0–117.0)		102.8 (16.4)		109.0 (94.0–118.0)		103.6 (15.1)		83.0 (77.0–92.0)		103.0 (13.1)	
G	137	106.0 (95.0–113.0)	0.082	102.1 (14.5)	0.597	103.0 (91.0–112.0)	**0.004**	100.8 (13.9)	0.113	86.0 (80.0–92.0)	0.239	100.1 (12.2)	0.097
A	67	108.0 (95.0–118.0)		103.3 (17.0)		109.0 (94.0–118.0)		104.2 (14.6)		83.0 (77.0–92.0)		103.2 (13.3)	
*NTRK2* rs2289656 (Dom)													
GG	70	108.0 (97.3–116.0)	0.260	103.3 (16.6)	0.406	106.0 (93.3–112.8)	0.651	102.7 (15.0)	0.436	84.5 (77.0–92.0)	0.560	101.7 (13.4)	0.456
AG + AA	32	104.5 (92.0–112.5)		100.6 (12.2)		106.0 (94.0–115.0)		100.3 (12.4)		87.5 (77.0–95.0)		99.7 (10.9)	
G	167	106.0 (95.0–116.0)	0.181	102.9 (15.9)	0.372	106.0 (94.0–112.0)	0.428	102.3 (14.6)	0.394	86.0 (77.0–92.0)	0.695	101.4 (13.0)	0.414
A	37	103.0 (92.0–112.0)		100.4 (12.1)		106.0 (94.0–115.0)		100.1 (12.0)		86.0 (77.0–95.0)		99.6 (10.8)	
*NTRK2* rs10868235 (Dom)													
GG	27	103.0 (93.0–113.0)	0.291	97.6 (11.5)	0.052	103.0 (91.0–112.0)	0.407	99.3 (11.7)	0.260	86.0 (77.0–95.0)	0.921	97.5 (11.3)	0.083
AG + AA	75	108.0 (95.0–116.0)		104.2 (16.2)		106.0 (94.0–115.0)		102.9 (15.0)		86.0 (77.0–92.0)		102.4 (12.9)	
G	106	106.0 (95.0–113.0)	0.405	100.8 (13.9)	0.096	106.0 (91.0–112.0)	0.453	101.1 (13.8)	0.409	86.0 (77.0–92.0)	0.875	100.0 (12.5)	0.201
A	98	108.0 (97.3–113.0)		104.3 (16.6)		106.0 (94.0–115.0)		102.8 (14.6)		86.0 (77.0–92.0)		102.3 (12.7)	

Dom: dominant model; Rec: recessive model. The bold indicates significant *p* values < 0.05. ^a^ Mann–Whitney test. ^b^ Student’s *t*-test.

**Table 4:** Influence of genetic polymorphisms on the cognitive profile assessed by WISC-V according to bisphenol exposure in children.

		Unadjusted Logistic Regression Models						Adjusted Logistic Regression Models						
		Low Exposure (≤Median)			High Exposure (>Median)			Low Exposure (≤Median)			High Exposure (>Median)			
SNP	Index	OR	95% CI	*p* Value	OR	95% CI	*p* Value	OR	95% CI	*p* Value	OR	95% CI	*p* Value	*p* for Interaction
*BDNF* rs11030101 (Ref. AA)														
AA vs. AT + TT (Dom)	VCI	0.29	0.08–1.02	0.053	0.91	0.28–2.89	0.869	0.18 ^d^	0.04–0.85	**0.031**	0.68 ^c^	0.18–2.59	0.575	0.302
Ref. A vs. T		0.49	0.22–1.08	0.078	1.19	0.53–2.70	0.672	0.26 ^d^	0.09–0.73	**0.011**	1.15 ^d^	0.50–2.64	0.738	0.067
*HTR2A* rs6314 (Ref. GG)														
GG vs. AG + AA (Dom)	VCI	0.34	0.08–1.48	0.150	0.35	0.08–1.62	0.180	0.15 ^d^	0.02–0.94	**0.042**	0.21 ^d^	0.03–1.33	0.098	0.820
Ref. G vs. A		0.32	0.08–1.29	0.109	0.33	0.08–1.35	0.122	0.22 ^d^	0.05–1.04	0.055	0.23 ^d^	0.04–1.22	0.084	0.946
*HTR2A* rs7997012 (Ref. AA)														
AA + AG vs. GG (Rec)	WMI	3.96	1.23–12.73	**0.021**	0.46	0.14–1.49	0.193	6.30 ^d^	1.38–28.73	**0.017**	0.27 ^d^	0.06–1.26	0.096	0.002 *
Ref. A vs. G		2.74	1.08–6.94	**0.033**	0.63	0.27–1.46	0.281	3.42 ^b^	1.22–9.53	**0.019**	0.49 ^d^	0.18–1.30	0.152	**0.007**
*MTHFR* rs1801133 (Ref. GG)														
GG vs. AG + AA (Dom)	WMI	0.28	0.09–0.91	**0.034**	0.75	0.21–2.67	0.657	0.24 ^c^	0.06–0.92	**0.038**	0.55 ^d^	0.11–2.78	0.467	0.272
Ref. G vs. A		0.31	0.13–0.73	**0.007**	1.18	0.52–2.69	0.689	0.28 ^c^	0.10–0.73	**0.010**	1.20 ^a^	0.49–2.93	0.686	**0.026**
*MTHFR* rs1801133 (Ref. GG)														
GG vs. AG + AA (Dom)	FSIQ	0.38	0.12–1.21	0.101	0.93	0.28–3.11	0.902	0.32 ^d^	0.08–1.29	0.111	0.68 ^d^	0.16–2.83	0.599	0.226
Ref. G vs. A		0.42	0.18–0.97	**0.041**	1.43	0.64–3.18	0.382	0.36 ^b^	0.14–0.91	**0.030**	1.43 ^a^	0.63–3.27	0.393	**0.025**
*OXTR* rs53576 (Ref. AA)														
AA + AG vs. GG (Rec)	FRI	0.49	0.15–1.61	0.238	0.26	0.08–0.86	**0.028**	0.69 ^d^	0.17–2.80	0.600	0.20 ^d^	0.05–0.78	**0.020**	0.315
Ref. A vs. G		0.74	0.29–1.91	0.531	0.53	0.23–1.26	0.152	0.99 ^d^	0.34–2.89	0.981	0.51 ^a^	0.21–1.21	0.126	0.370
*OXTR* rs53576 (Ref. AA)														
AA + AG vs. GG (Rec)	WMI	0.91	0.30–2.74	0.869	0.24	0.07–0.80	**0.021**	1.08 ^d^	0.29–4.02	0.905	0.08 ^d^	0.01–0.50	**0.007**	**0.030**
Ref. A vs. G		0.97	0.40–2.31	0.937	0.42	0.17–1.07	0.070	1.14 ^d^	0.43–3.04	0.787	0.27 ^d^	0.09–0.83	**0.023**	0.066
*SLC6A2* rs998424 (Ref. GG)														
GG vs. AG + AA (Dom)	FRI	1.68	0.50–5.66	0.403	0.18	0.05–0.60	**0.006**	2.14 ^d^	0.53–8.64	0.285	0.16 ^c^	0.04–0.57	0.005 *	0.004 *
Ref. G vs. A		1.36	0.58–3.20	0.476	0.30	0.13–0.71	**0.006**	1.35 ^a^	0.56–3.26	0.500	0.26 ^c^	0.11–0.65	0.004 *	0.004 *
*SNAP25* rs363039 (Ref. GG)														
GG vs. AG + AA (Dom)	FRI	0.62	0.19–2.02	0.430	0.19	0.06–0.68	**0.010**	0.55 ^b^	0.16–1.94	0.353	0.17 ^b^	0.04–0.63	**0.008**	0.124
Ref. G vs. A		0.58	0.24–1.40	0.226	0.27	0.11–0.64	**0.003**	0.45 ^d^	0.16–1.26	0.128	0.28 ^a^	0.12–0.68	0.005 *	0.258
*SNAP25* rs363039 (Ref. GG)														
GG vs. AG + AA (Dom)	WMI	0.41	0.13–1.27	0.124	0.56	0.17–1.86	0.344	0.36 ^b^	0.11–1.19	0.094	0.29 ^d^	0.07–1.27	0.099	0.859
Ref. G vs. A		0.53	0.23–1.24	0.144	0.51	0.22–1.17	0.112	0.43 ^b^	0.17–1.09	0.075	0.33 ^d^	0.11–0.95	**0.040**	0.775
*SNAP25* rs363039 (Ref. GG)														
GG vs. AG + AA (Dom)	FSIQ	0.29	0.09–0.92	**0.035**	0.41	0.13–1.33	0.137	0.19 ^d^	0.05–0.82	**0.026**	0.28 ^d^	0.07–1.09	0.067	0.820
Ref. G vs. A		0.42	0.18–0.99	**0.047**	0.57	0.25–1.30	0.181	0.26 ^d^	0.09–0.78	**0.016**	0.59 ^a^	0.25–1.37	0.221	0.378
*NTRK2* rs2289656 (Ref. GG)														
GG vs. AG + AA (Dom)	VCI	3.43	0.99–11.93	0.053	0.96	0.29–3.24	0.951	9.06 ^c^	1.51–54.39	**0.016**	0.96 ^d^	0.23–3.91	0.951	0.088
Ref. G vs. A		2.97	1.05–8.44	**0.041**	1.11	0.38–3.27	0.844	6.72 ^d^	1.82–24.83	0.004 *	0.89 ^b^	0.28–2.82	0.837	0.062
*NTRK2* rs10868235 (Ref. GG)														
GG vs. AG + AA (Dom)	VCI	0.26	0.07–0.98	**0.046**	0.79	0.21–2.95	0.730	0.22 ^d^	0.04–1.08	0.062	2.09 ^d^	0.41–10.72	0.377	**0.043**
Ref. G vs. A		0.53	0.24–1.17	0.117	0.93	0.42–2.04	0.854	0.46 ^b^	0.19–1.13	0.090	1.40 ^d^	0.58–3.37	0.458	0.094
*NTRK2* rs10868235 (Ref. GG)														
GG vs. AG + AA (Dom)	VSI	0.31	0.08–1.30	0.110	1.00	0.27–3.66	1.000	0.18 ^d^	0.04–0.88	**0.034**	5.35 ^d^	0.60–47.42	0.132	**0.020**
Ref. G vs. A		0.92	0.41–2.06	0.840	1.08	0.49–2.37	0.841	0.66 ^b^	0.27–1.62	0.362	1.56 ^b^	0.61–4.03	0.357	0.199

Ref: reference category; Dom: dominant model; Rec: recessive model. Bold indicates significant *p* values < 0.05, and the asterisk (*) means significant *p* values after Bonferroni’s correction (*p* < 0.005). ^a^ Adjusted for gender and age. ^b^ Adjusted for gender, age, and BMI. ^c^ Adjusted for gender, age, and parental education level. ^d^ Adjusted for gender, age, BMI, and parental education level.

## Data Availability

Data are contained within the article.

## Supplementary Materials

The following supporting information can be downloaded at https://www.mdpi.com/article/10.3390/nu16162639/s1, Table S1. Influence of genetic polymorphisms on the cognitive profile of children assessed by WISC-V according to bisphenol exposure.
